# Microseconds Simulations Reveal a New Sodium-binding Site and the Mechanism of Sodium-coupled Substrate Uptake by LeuT*

**DOI:** 10.1074/jbc.M114.617555

**Published:** 2014-11-07

**Authors:** Elia Zomot, Mert Gur, Ivet Bahar

**Affiliations:** From the Department of Computational & Systems Biology, School of Medicine, University of Pittsburgh, Pittsburgh, Pennsylvania 15213

**Keywords:** Biophysics, Membrane Transport, Neurotransmitter Transport, Sodium Transport, Transport, LeuT, NSS, Mechanism of Binding, Sodium-coupled

## Abstract

The bacterial sodium-coupled leucine/alanine transporter LeuT is broadly used as a model system for studying the transport mechanism of neurotransmitters because of its structural and functional homology to mammalian transporters such as serotonin, dopamine, or norepinephrine transporters, and because of the resolution of its structure in different states. Although the binding sites (S1 for substrate, and Na1 and Na2 for two co-transported sodium ions) have been resolved, we still lack a mechanistic understanding of coupled Na^+^- and substrate-binding events. We present here results from extensive (>20 μs) unbiased molecular dynamics simulations generated using the latest computing technology. Simulations show that sodium binds initially the Na1 site, but not Na2, and, consistently, sodium unbinding/escape to the extracellular (EC) region first takes place at Na2, succeeded by Na1. Na2 diffusion back to the EC medium requires prior dissociation of substrate from S1. Significantly, Na^+^ binding (and unbinding) consistently involves a transient binding to a newly discovered site, Na1″, near S1, as an intermediate state. A robust sequence of substrate uptake events coupled to sodium bindings and translocations between those sites assisted by hydration emerges from the simulations: (i) bindings of a first Na^+^ to Na1″, translocation to Na1, a second Na^+^ to vacated Na1″ and then to Na2, and substrate to S1; (ii) rotation of Phe^253^ aromatic group to seclude the substrate from the EC region; and (iii) concerted tilting of TM1b and TM6a toward TM3 and TM8 to close the EC vestibule.

## Teaser

**Background:** LeuT is a homologue of neurotransmitter transporters.

**Results:** Microseconds simulations disclose multiple translocation sites for Na^+^, including a new site for initial binding.

**Conclusion:** Coupled Na^+^- and substrate-binding events are accompanied by local and global (interhelical) rearrangements in the outward facing structure.

**Significance:** New insights are gained into Na^+^ and substrate uptake/efflux mechanisms of LeuT.

## Introduction

Neurotransmitter:sodium symporters (NSSs)^3^ are involved in many neurological disorders including epilepsy (1), depression (2, 3), anxiety (4, 5), and Parkinson's (5) disease and are targets of both clinical and illicit drugs, including stimulants such as cocaine and amphetamine and antidepressants such as fluoxetine (6–8). NSSs carry out their function by coupling the energetically unfavorable translocation of their substrate across the cell membrane to the transport of ions, namely sodium ions (9, 10), down their electrochemical gradient. Neurotransmitter import by NSSs is coupled to that of 1–3 sodium ions and may involve the co-transport or countertransport of other ions such as chloride (10–13).

**FIGURE 1.**
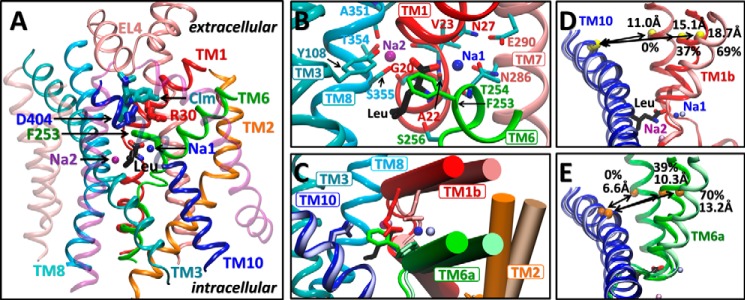
**Structure, binding sites, and conformational changes in LeuT.** *A*, LeuT monomeric unit in OF-occluded form (PDB code 3F3E), viewed from the side. Leu (in *CPK sticks*; carbons in *black*) is shielded from the EC region by Phe^253^ side chain (*green*) on TM6. Na^+^ ions at sites Na1 and Na2 are shown in *blue* and *purple*, respectively. The secondary binding site (S2) binds larger noncompetitive inhibitors such as clomipramine (*Clm*, *sticks*, *teal*) (PDB code 2Q6H). *B*, a close up on the coordination of Leu and Na^+^ ions as viewed from the EC space. *C*, superposition of OF-occluded and -open (PDB code 3TT1) structures (*darker* and *lighter colors*, respectively), showing the motions in TM1b, 2 and 6a (*cylinders*), the rotation of Phe^253^ side chain, and the displacement of Arg^30^ (*dark/light red*) away from Asp^404^ (*dark/light blue*). *D* and *E*, changes in distances between Val^33^(C) and Asp^401^(C^α^) (*D*, *yellow spheres*) and Ile^245^(C) and Ile^410^(C^α^) (*E*, *orange spheres*), used as probes for TM1b–TM10 and TM6a–TM10 separations, respectively. The distances in the crystal structures used in this study are indicated, along with their relative openings with respect to the experimentally resolved inward facing occluded state (0%) and the maximal opening (100%) observed in simulations.

The sodium:leucine transporter from *Aquifex aeolicus* (LeuT), a bacterial NSS, has served as a model for NSSs because of the early determination of its high resolution structures in various states (14–17). These structures have shown that LeuT is a homodimer, with the monomeric unit composed of twelve transmembrane (TM) helical domains (Fig. 1*A*). Ten of them obey an internal pseudosymmetry whereby the first five (TM1–TM5) can be superposed onto the second five (TM6–TM10) by a rigid body rotation of ∼180° (16, 18). Another notable feature of the LeuT fold is the disruption of the α-helical geometry in the first helix of these two substructures (TM1 and TM6), approximately halfway across the membrane at the so-called transport core, exposing carbonyl and amide groups that bind the substrate and two sodium ions.

The first LeuT structure was resolved in an outward facing (OF) form in the presence of substrate and two Na^+^ ions (16). Leu-bound structure and those resolved with other substrates, such as alanine or glycine are highly similar (backbone root mean square deviation of ∼ 0.3 Å) (15). In all cases, the substrate is bound in a pocket formed by amino acids on TM1, TM3, TM6, and TM8 (the primary or S1 site). Its carboxylate group interacts with the Na^+^ ion located at site Na1. It is ∼6 Å away from the second Na^+^ (bound to site Na2) (Fig. 1*B*). The overall (global) structure of LeuT is OF, but the primary site is occluded by the side chain of Phe^253^ on TM6, which shields the substrate from the extracellular (EC) environment. Phe^253^ is referred to as the “thin” gate, as opposed to the “thick” gate secluding S1 from the intracellular region.

Open forms of LeuT were resolved in the absence of substrate (with Na^+^ only) (14) or with a competitive inhibitor (tryptophan) at S1 (15). The OF-open form is distinguished from the OF-occluded mainly by ∼10° reorientations in the EC halves of TM1 (TM1b), TM2, and TM6 (TM6a) away from the center and by a ∼90° rotation in Phe^253^ side chain dihedral χ_1_ (Fig. 1, *C–E*), which expose the binding site to the EC region (14, 15). Another distinctive feature is the disruption of the direct or water-mediated interaction between Arg^30^ (TM1) and Asp^404^ (TM10), which otherwise form another EC gate ∼8 Å above the site S1 (Fig. 1*C*).

In addition to the primary site S1 for substrate binding, a secondary site (S2) has been reported in the EC vestibule ∼10 Å above S1 (17, 19), which binds detergents (20) or noncompetitive inhibitors such as tricyclic antidepressants (19) (*e.g.* desipramine and clomipramine Clm) (Fig. 1*A*). The issue of whether S2 serves as a high affinity substrate-binding site that impacts transport function has been a subject of controversy among leading groups in the field: studies mainly by the Weinstein and Javitch (21–23) laboratories supported that S2 is a high affinity substrate-binding site with functional significance; those by Gouaux and coworkers (15, 16, 24–26) supported that there is only a single high affinity site (S1) in LeuT.

Even though the binding sites and poses for the Na^+^ ions and substrate have been crystallographically resolved, their coupled pathways of entry, binding, and translocation remain to be elucidated. A wide range of turnover rates have been reported for neurotransmitter transporters, varying from 1 to 15 per s (27), to *k*_cat_ of 0.1 to 0.65 per min for uptake by LeuT (24), *i.e.* the transport cycle takes hundreds of milliseconds, if not minutes. Full atomic simulation of molecular events at this time scale is beyond the capability of current computing technology, unless approximate models or methods are resorted to. We chose to adopt a full atomic and unbiased approach and therefore focused on substrate and Na^+^ uptake events and accompanying transitions between the occluded and *open* forms of the OF LeuT. These events take place on much shorter time scale (nano- to microseconds). With the help of simulations of over 20 μs that are pushing the boundaries of current full atomic molecular dynamics (MD) simulation technology, we thoroughly examined the sequence of substrate- and Na^+^-binding/unbinding events and the accompanying conformational changes.

To our knowledge, these are the most extensive MD simulations performed to date for LeuT dimer, and they generate for the first time trajectories capable of exhibiting the coupled dynamics of substrate and cations during substrate binding (and unbinding) in the OF state. They also provide robust information on new transient or functional binding sites for substrate and cations.

## EXPERIMENTAL PROCEDURES

### 

#### 

##### Structure Preparation

The OF-occluded LeuT structure co-crystallized with one Leu and two Na^+^ ions (PDB code 3F3E) was used as the initial structure for simulating the occluded WT symporter and mutants K288A and Y108F in the absence (runs 3 and 4) or presence (runs 7–9) of ions and substrate (Table 1). This structure is highly similar to the occluded form crystallized with other substrates including alanine and methionine (backbone root mean square deviation ≈ 0.3 Å). The OF-open structure adopted as initial structure here is that of the double mutant K288A/Y108F crystallized in the presence of two Na^+^ ions (runs 1, 2, 5, and 6) (PDB code 3TT1), which is highly similar (backbone root mean square deviation ≈ 0.5 Å) to LeuT resolved in the presence of tryptophan (PDB code 3F3A).

**TABLE 1:** **Initial simulation conditions, durations, and identifiers of the runs performed** Each of the 15 productive runs was performed on dimeric LeuT (WT or mutant), initiated after total equilibration of 50–70 ns, during which constraints on the backbone and substrate/Na^+^ (when present) were gradually lifted. Substrate/Na^+^ and/or LeuT mutations were inserted into (runs 3 and 9) or removed from (run 2) the equilibrated structure (over 30–50 ns) prior to a final 20-ns equilibration step. See Table II for the changes (binding/unbinding events and TM1/TM6 opening/closing) observed in the runs.

Run ID	Initial state	Initial PDB structure	LeuT sequence	Initially bound substrate/ions	Simulation duration
					μ*s*
1	OF-open	3TT1	K288A/Y108F	2Na^+^	1.0
2			WT		1.0
3a–3b	OF-occluded	3F3E	K288A	None	1.0 (a), 1.0 (b)
4			WT		1.0
5	OF-open	3TT1	WT	Ala/2Na^+^	0.5
6a–6c				Leu/2Na^+^	1.5 (a), 1.5 (b), 1.0 (c)
7	OF-occluded	3F3E	WT	Ala/2Na^+^	2.0
8a–8c				Leu/2Na^+^	3.0 (a), 3.0 (b), 1.5 (c)
9a–9b			Y108F	Leu/2Na^+^	0.5 (a), 1.4 (b)

Simulations were performed for LeuT dimer, given that NSS members usually exist as dimers or oligomers (28–32). Each dimer was embedded in a 1-palmitoyl-2-oleoyl-phosphatidylethanolamine (POPE) membrane bilayer with dimensions of 155 × 110 Å^2^ and a solution with 150 mm NaCl. Water (TIP3P) molecules and POPE head groups were modeled explicitly, whereas the united atom model was used for POPE acyl chains (33). Each run contained ∼118,000 atoms, consisting of ∼16,000 in the transporter, ∼20,000 in the membrane, and ∼82,000 for water, and ∼164 ions (77 Na^+^ and 87 Cl^−^). Equilibrated systems had at least 19 Å of lipid padding along the *x*/*y* axes or 18 Å of water along the *z* axis. All titratable residues were in their dominant protonation state at pH 7.

##### Simulation Protocols

All simulations were performed using the CHARMM36 force field (34, 35). Each structure was equilibrated with harmonic constraints on the protein backbone and on substrate/Na^+^ atoms (using force constants of 10 kcal·mol^−1^·Å^−2^ for 10 ns, followed by 4 kcal·mol^−1^·Å^−2^ for 20 ns). After this initial equilibration step, introduction of mutations (runs 3 and 9) or reversion to WT (runs 2, 5, and 6), and addition or removal of substrate/Na^+^ (runs 3–7) were performed *in silico* (Table 1). Every system was then neutralized, minimized, and run for an additional 20 ns with lower constraints (force constant of 2 kcal·mol^−1^·Å^−2^ on the same atoms) to allow adequate adaptation to the introduced changes. Productive runs of 20 ns each were performed using NAMD-2.8 (36) followed by the 512-node Anton machine (37) as listed in Table 1. A time step of 2 fs was used for short range electrostatic and van der Waals interactions (cutoff distance of 12 Å) and of 4 fs for long range electrostatics using the particle mesh Ewald method. All runs were conducted under constant temperature and pressure conditions at 1 bar and 310 K, using the Berendsen coupling scheme. Trajectory visualization and analyses were performed with VMD-1.9.1 (38).

## RESULTS

### 

#### 

##### In the Outward Facing Open Form of LeuT, Sodium Is Released from the Na2 site, but Not Na1, into the EC Environment

**FIGURE 2.**
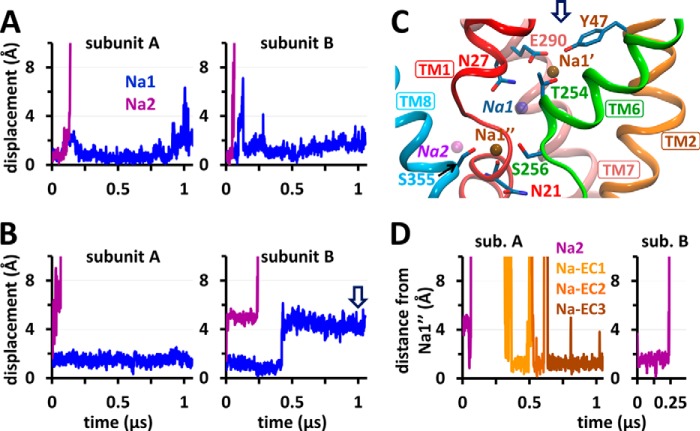
**Sodium unbinding and binding events in the OF-open state of LeuT dimer in the absence of substrate and identification of a novel Na^+^-binding site Na1″.** *A* and *B*, displacements of sodium ions originally located at Na1 and Na2 (*blue* and *purple*, respectively) away from their crystal positions, presented for subunits A and B in double mutant Y108F/K288A (*A*) and WT-LeuT (runs 1 and 2, respectively; Table 1) (*B*). *C*, a snapshot of subunit B of WT-LeuT (run 2) at ∼1 μs (*arrow* in *B*) shows the locations of two sites, Na1′ and Na1″, observed to stably bind sodium ions, and the vacated Na1 and Na2 sites (*transparent blue* and *purple*, respectively). The sodium ion at Na1′ (originally at Na1) is coordinated by Glu^290^ (TM7) and polar residues (*sticks*). Na^+^ at Na1″ is coordinated by Asn^21^, Ser^256^, Ser^355^, and Asn^286^ (omitted for clarity). *D*, high propensity of Na1″ site, 5–6 Å away from Na2, for binding Na^+^ ions, either on their way out to the EC environment (for Na^+^ ions originally bound to Na2; *purple*) or coming in from the EC environment (*Na-EC1*, *Na-EC2*, or *Na-EC3*, *orange-brown*). The data in *D* refer to run 2 (*B*). *sub.*, subunit.

We first explored the binding/unbinding of Na^+^ ion in the absence of substrate (runs 1–4; Table 1). The OF-open LeuT structure crystallized with sodiums bound to Na1 and Na2 sites, adopted in run 1, was stabilized by the Y108F/K288A double mutation, where Y108F abolishes the binding of Leu at site S1 and K288A retains a WT-like behavior while facilitating the solubilization and crystallization of the transporter (14, 24). The sodium ions at the Na2 site of both subunits were observed to completely dissociate within ∼0.12 μs, via the EC vestibule. In contrast, those at Na1 remained bound over microsecond simulations (Fig. 2*A*). Similar behavior was observed when WT residues were restored *in silico* (run 2): unbinding from Na2 took place in both subunits within 0.25 μs, whereas the Na^+^ ion at Na1 never left into the EC vestibule/solution but either remained bound or migrated to a neighboring location ∼5 Å away from Na1 (Fig. 2*B*, *arrow*). At this site, designated as *Na1′* (39), the carboxylate of Glu^290^ (TM7) together with the side chain carbonyl/amide of Asn^27^ (TM1) and Asn^286^ (TM7), and hydroxyls of Tyr^47^ (TM2) and/or Thr^254^ (TM6) coordinate Na^+^ (Fig. 2*C*, *arrow*). A color-coded summary of successive translocation sites of Na^+^ ions originally bound to Na1 and Na2 is given in Table 2 (runs 1 and 2).

**TABLE 2:**
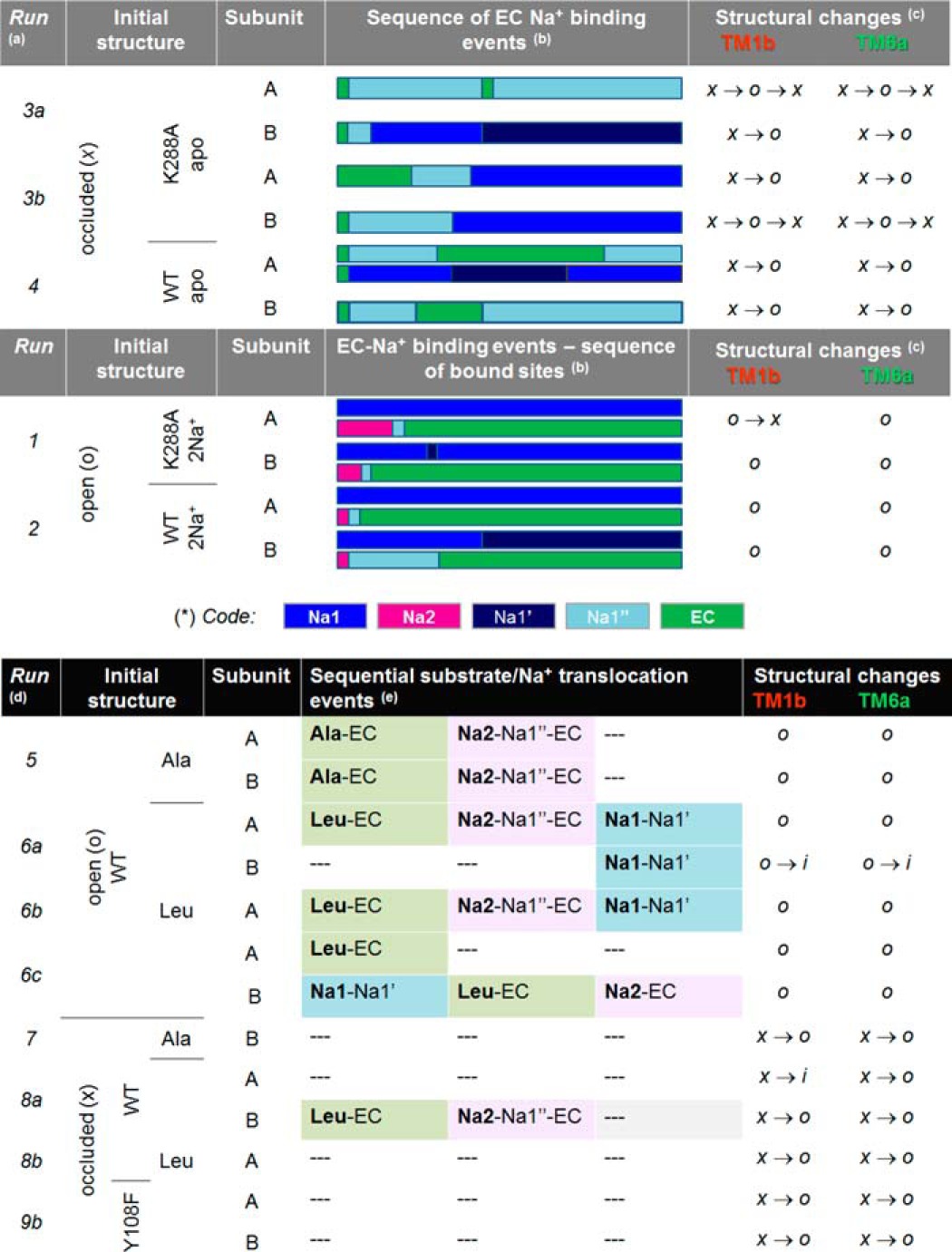
**Sequence of sodium-binding/unbinding (top) and coupled substrate/Na^+^ dislocation (bottom) events and accompanying changes in TM1b and TM6a**

*^a^* The upper portion of the table describes the events in the absence of substrate, the lower describes the events in its presence. See also Table 1.

*^b^* Runs 3 and 4 describe Na^+^ binding events, runs 1 and 2 describe Na^+^ unbinding events. The color-coded bars provide qualitative information on the trajectory of the Na^+^ ions in each subunit (color code at the bottom). In each case, the initial position of Na^+^ ions (EC, runs 3 and 4; or Na1- and Na2-bound, runs 1 and 2) is shown, along with instances of binding that last at least 50 ns. If a subunit simultaneously binds more than one Na^+^ ion, a pair of bars are shown (*e.g.* subunit A in run 4; all subunits in runs 1 and 2).

*^c^* TM1b/6a are regarded as open (o) if their percent opening (Fig. 1*D* and *E*) is larger than 60%, intermediate (i) if in the range 50–60%, and occluded (x) if less than 50%. x → o indicates a transition from occluded to open; x → 0 → x indicates an opening followed by a closing. Complete opening of EC vestibule takes place when both TM1b and TM6a undergo the x → o transition (*e.g.* subunit B in run 3a, and both subunits in runs 4, 8b and 9b.

*^d^* Only those runs and subunits where either the substrate or cations showed a dislocation or TM1b/6a helices underwent a structural change are listed.

*^e^* Sequence of events listed from left to right, based on observed trajectories for each subunit, starting by the identity of substrate/ion that undergoes movement (AIa, Leu, or Na1- or Na2-bound sodium), written in bold type followed by sites/regions (*e.g.* Na1′, Na1″, and EC) they sequentially visit. In run 6b subunit A, Leu and the sodium at Na2 dislocated almost simultaneously.

These sodium unbinding events indicate that the Na1 site has a higher affinity for sodium than does Na2 in the OF-open form. We will see below that this tendency is maintained in the presence of a substrate (Leu or Ala) bound to S1: in five of eight examined subunits (runs 5 and 6a–6c), sodium migrated away from Na2 and either remained within the confines of the EC vestibule (two cases) or left entirely into the EC region (three cases) (Table 2). In contrast, sodium bound at Na1 never migrated to the EC vestibule but either remained bound over the entire simulation time (four cases, each 1–1.5 μs long) or translocated to Na1′ to either remain stably bound to that site, or return to Na1 (another four cases; Table 2). In summary, based on ten independent observations (five runs, two subunits per run) of substrate-free or -bound WT-LeuT in the OF-open state, we conclude that Na1-bound sodium exhibits equal probability of either remaining bound to Na1 or migrating to Na1′, whereas that bound to Na2 dissociates and either remains in the EC vestibule or escapes to the EC region.

##### Sodium Binding to Na1′ Consistently Follows a First Binding to Na1

The Na1′ site has been proposed, using umbrella sampling, to act as a transient Na^+^-binding site that facilitates the uptake of Na^+^ from the EC environment and succeeding binding the Na1 site (39). Our unbiased simulations show, on the other hand, that binding of Na^+^ ions to Na1′ was consistently preceded by their binding to Na1 (Table 2, runs 5 and 6). Sodium ions dislodged from Na1 would move between the Na1 and Na1′ sites, but not diffuse into the EC region, and those binding from the exterior would first bind Na1 (as explained below) and then Na1′. Examples for stable Na1′ binding consistently observed in our simulations are displayed in Figs. 2*B* (run 2) and 3*C* (run 3a).

**FIGURE 3.**
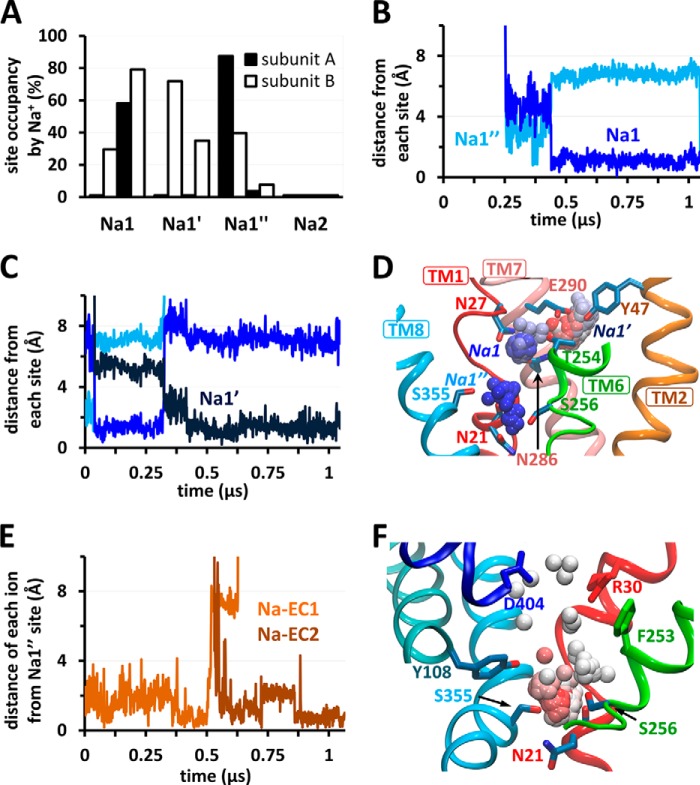
**Time-dependent occupancy of sodium-binding sites Na1, Na1′, and Na1″ and sequential binding events as the OF-occluded K288A mutant transitions into OF-open state.** *A*, in the absence of substrate and sodium ions (runs 3a and 3b), EC sodium ions enter the EC vestibule of K288A mutant and bind to the sites Na1, Na1′, and Na1″, but not to Na2, as shown by the percentage occupancies of these sites by Na^+^. *B*, the instantaneous position of an EC Na^+^ ion that enters subunit A (run 3b), using as metric its distances from the sites Na1″ (*blue*) and Na1 (*dark blue*). The EC Na^+^ ion first comes into close proximity of Na1″ and then binds Na1 where it settles for the remaining 0.6 μs of simulation. *C*, the behavior of another EC Na^+^ ion (subunit B; run 3a), which first recognizes and momentarily binds at Na1″ and then switches to Na1 (*blue*) and Na1′ (*dark blue*). *D*, the trajectory in *C* is illustrated where the *spheres* display the instantaneous positions of EC Na^+^ sampled at 1.25-ns intervals during 0 < *t* < 1 μs (color-coded by time from *blue* to *red*). *E*, stable binding of two EC Na^+^ ions (*orange* and *brown*) to Na1″ in subunit A (run 3a). *F*, the trajectory of the second EC Na^+^ ion (*Na-EC2*) in *D* is shown here for the time period 0.5 < *t* < 1 μs. On-pathway gating and/or Na^+^-coordinating residues are shown in *sticks*.

In contrast to eukaryotic transporters, uptake activity in LeuT and other prokaryotic NSS is independent of chloride (41, 42). The negatively charged carboxylate of Glu^290^, observed here to coordinate sodium at Na1′ (Figs. 2*C* and 3*D*) has been shown to fulfill the role of a chloride ion that binds at the equivalent position when a neutral residue is present as in mammalian NSS (43–47). In addition, an acidic residue at this position was found to couple substrate/Na^+^ symport to proton antiport: after sodium is released into the cytoplasm, Glu^290^ can be protonated, and this neutralization of charge facilitates the return of the unloaded transporter to the OF form (47, 48). Our observations support a functional role for Glu^290^ in LeuT.

##### A Newly Identified Site, Na1″, near Asn^21^, Forms the Entry Route for EC Sodium Binding

Whereas the Na1′ site was able to capture the sodium leaving Na1, another location had an even higher overall occupancy by sodium. This newly discovered site, which we refer to as Na1″ (Figs. 2*C* and 3, *D* and *F*), is located in the cavity otherwise occupied by the substrate in S1, ∼5–6 Å away from both Na1 and Na2. It is coordinated by Ser^256^ (TM6) and Ser^355^ (TM8) hydroxyls and by Asn^21^ (TM1) backbone carbonyl, and often the side chain amide as well.

Notably, Na1″ invariably serves as an attractor for Na^+^ ions entering from the EC medium (Table 2, runs 3 and 4). The same path of entry, via Na1″, was consistent for the three Na^+^ ions that entered LeuT from the exterior (run 2; Fig. 2*D*). Furthermore, Na^+^ ions leaving Na2, runs 1 and 2, either passed transiently through or stably bound the Na1″ site (Table 2).

##### Extracellular Sodium Migrates from Na1″ to Na1 Site in the OF-occluded apo Transporter

**FIGURE 4.**
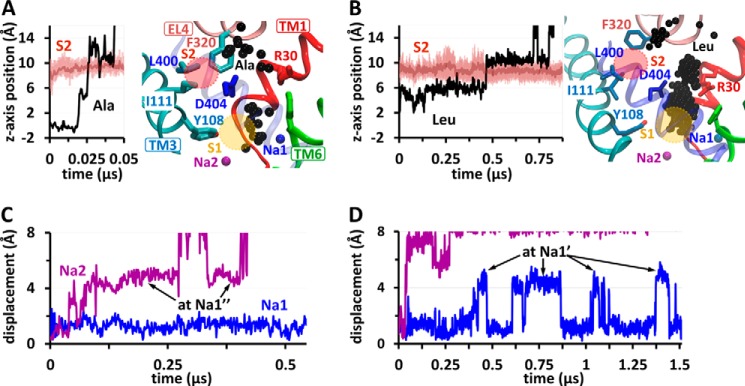
**Substrate translocation to the EC environment in OF-open form and accompanying movements of sodium ions.** *A* and *B* illustrate the translocation of Ala (run 5, subunit A) and Leu (run 6a, subunit A), respectively. The *left panels* show the instantaneous position of substrate along the *z* axis (perpendicular to membrane) (*black curve*), along with those of the center and upper/lower boundaries of the S2 site (*dark pink* and *pink curves*, respectively). The S1 site is at *z* = 0. The substrate moves “upwards” into the EC region. The *right panels* illustrate the successive positions (*black spheres*) of Ala or Leu (C^α^-atoms), along the pathway to the EC region as viewed from the side sampled at 1.25ns. S1 and S2 sites are shown (*yellow* and *pink circles*, respectively). Residues that define the sites S1 (Tyr^108^; TM3) and S2 (Ile^111^ (TM3), Leu^400^ (TM10), and Phe^320^ (EL4)) and the EC gating residues Phe^253^ (*green*), Arg^30^ (*red*), and Asp^404^ (*blue*) are shown in *sticks*. The accompanying displacements of Na^+^ ions away from their original locations at Na1 and Na2 are shown in *C* and *D*, respectively. The *arrows* indicate the temporary bindings from Na2 to Na1″ (*C*) or from Na1 to Na1′ (*D*).

With the rationale that the order of unbinding events can be deduced from the reverse of that of binding, we explored sodium binding upon the return of the inward facing (IF) unloaded transporter en route to the OF-open form. We removed the bound leucine and two sodium ions in the OF-occluded form and examined occupancy of both Na1 and Na2, as well as of other locations where repeated sodium binding events were observed including Na1′ and Na1″. In both K288A-LeuT (runs 3a and b) and WT-LeuT (run 4), EC sodium that entered the EC vestibule invariably reached the Na1 site through the new site Na1″. Fig. 3*A* displays the fractional occupancy of all four sites, Na1, Na2, Na1′, and Na1″, observed in runs 3a and 3b. EC sodium spontaneously bound to Na1 in ∼40% of the total time for all four subunits in K288A-LeuT (Fig. 3*A*) and 35% for the two in WT-LeuT (run 4; not shown). In contrast, no sodium ions entered the Na2 cavity in any run, further demonstrating the low affinity of Na2 in the OF state. Sodium ions entered the external cavity mostly via interaction with or in proximity to Asp^404^ on TM10 (Fig. 3*F*), bound to Na1″ transiently (Fig. 3, *B* and *C*) or stably (Fig. 3*E*) before reaching the Na1 site where they remained bound for at least hundreds of nanoseconds either till the end of the simulations (Fig. 3*B*) or prior to dislocation to Na1′ (Fig. 3*C*) consistent with the sequence of events observed in the open state. After binding Na1′, sodium either remained bound there (Figs. 2*B*, subunit B, and 3*C*) or returned to Na1 (Fig. 4*D* and see below) but never left to other sites. In one exception, EC sodium entered the Na1 site without binding transiently at Na1″, but only because the latter was already occupied by another Na^+^ ion. In contrast to sodium, no chloride ions entered the EC cavity in any of the subunits, regardless of the starting conformation or the occupancy state of the substrate/sodium binding sites (runs 1–6).

##### Binding of EC Na^+^ to Na1″ Site Is Succeeded by Opening of the EC Vestibule

To examine the change in LeuT conformation triggered by EC Na^+^ binding (runs 3a, 3b, and 4), we measured the extent of opening/closure of the EC vestibule. We chose as probes of TM1b–TM10 and TM6a–TM10 interhelical distances the respective backbone atom pairs Val^33^(C)-Asp^401^(C^α^) and Ile^245^(C)-Ile^410^(C^α^) where maximal difference is observed between the external transport and scaffold domains after aligning the IF-open, OF-open, and OF-occluded forms (Fig. 1, *D* and *E*). These points, all of which lie approximately along the same reaction coordinate upon aligning the three structures, provide a reasonable measure of the separation between the mobile TM1b or TM6a and the relatively stable TM10 that lies across the EC cavity. Interestingly, our simulations further indicated that the degree of opening in the EC domains seen in the crystal structures does not represent the maximal degree of opening. Data from all runs suggest that the distances seen in the experimentally resolved OF-occluded and -open structures represent ∼40 and ∼70%, respectively, of the maximal (100%) opening of the vestibule observed in simulation, using the IF state as a reference for maximal closure (0%) (Fig. 1, *D* and *E*).

All subunits of the initially occluded LeuT with Leu at S1, both K288A and WT, were able to reach a degree of opening similar to or exceeding that of the open form within 0.5 μs upon binding of EC Na^+^ ions. The EC Na^+^ ion located at Na1″ was attracted by Asp^404^ (TM10) at the equilibration stage or shortly after the initiation of the productive run. The EC vestibule was mostly or completely closed at the initial binding stage. Binding to Na1″ was shortly followed by, if it did not trigger, the opening of the EC vestibule, which was accompanied by binding to the deeper site Na1 (three of four cases). Notably, in five of six cases, one or more of the Na1, Na1′, and Na1″ sites had a Na^+^ ion bound most of the time, and the EC vestibule remained open till the end of simulation in four of six subunits (Table 2). In contrast to the important effect of EC sodium binding to Na1″/Na1/Na1′ on LeuT conformation, sodium at Na2 had little or no effect on LeuT conformation when released to the EC region in the absence (runs 1 and 2) or presence of substrate (runs 5, 6a, and 6b, discussed below).

##### Substrate Dissociation in the OF State Precedes That of Sodium at Na2 and Does Not Involve Binding to S2

Because LeuT can transport several amino acids in addition to Leu, such as Ala and Gly (15), and for a more comprehensive investigation of the correlation between sodium and substrate binding/dissociation, we simulated the dynamics of the OF-open state in the presence of Na^+^ ions with the S1 site occupied by either leucine or alanine. LeuT binds Ala with a lower affinity than Leu (respective K*_d_* values of ∼500 and 20 nm) but translocates it with a 5-fold higher turnover rate (respective *k*_cat_ values of 6.1 versus 1.2 h^−1^) (15). In addition to simulating the occluded OF-form with the substrate (discussed below), we have also looked into the dynamics of Leu or Ala placed in the OF-open form. This was performed for two reasons in support of substrate binding to the open rather than occluded state: the higher probability of substrate entrance into a more accessible and hydrated cavity and the fact that binding of sodium can precede that of the substrate given that sodium binds and/or stabilizes the open form as shown above.

The Ala-bound form was constructed based on Leu-bound crystal structure, upon mutating the side chain of leucine *in silico*. Ala was observed to dislocate into the EC environment in both subunits within tens of nanoseconds at most (Fig. 4*A*, run 5), consistent with the low affinity of the OF-open state for Ala. This process was accompanied by little or no binding to the site S2 or any other secondary site within the EC vestibule.

**FIGURE 5.**
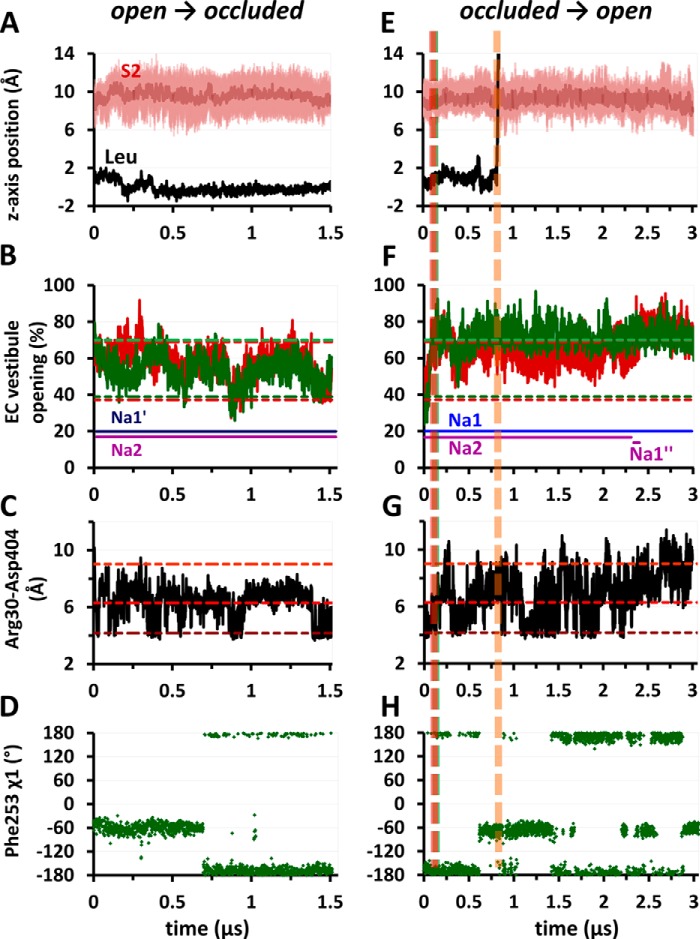
**Gating and structural changes associated with binding and release of leucine in the OF state.** Forward (*A–D*) and reverse (*E–H*) transitions between open and occluded forms, accompanying the respective binding or unbinding of leucine are shown. *A* and *E*, instantaneous position of Leu along the *z* axis (*black*) with respect to S1 site (at *z* = 0) and that of the S2 site (*dark/light pink*). *B* and *F*, the degree of opening of TM1b and 6a (*red* and *green*, respectively), as well as the occupancy of Na1, Na2, Na1′, or Na1″ sites by sodium ions initially at Na1 or Na2 (*blue* or *purple*, respectively) for each subunit. *C* and *G*, distance between Arg^30^:Cζ and Asp^404^:Cγ. The *dashed lines* in *dark*, *medium*, and *light red* indicate the crystal structure values for the direct, water-mediated, or no interactions (4.2, 6.3, or 9.0 Å, respectively) observed in IF-open, OF-occluded, and OF-open states. *D* and *H*, time evolution of Phe^253^ χ_1_ dihedral. The average values −67° and −164° correspond to the OF-open and -occluded crystal structures, respectively. *E–H*, *vertical dashed lines* indicate the times of TM1b opening (*red*), TM6a opening (*green*, overlaps with *red line*), and substrate unbinding from S1 (*orange*). The data are shown for subunit B in run 6a (*A–D*) and *B* in run 8a (*E–H*).

Leu also tended to leave the transporter into the EC environment (in four of six cases, runs 6a–6c), as shown in Fig. 4*B*. However, whereas Ala dislocation from S1 and complete migration to the EC region took place within 50 ns, complete departure of Leu varied over a broader range of time (up to 1 μs), and in two cases Leu remained mostly within the confines of S1 site over the entire duration of simulations (Fig. 5*A*). In two of four unbinding events, Leu left LeuT with little or no binding in the EC vestibule above S1, including the S2 region, whereas in the other two, it was detained for hundreds of nanoseconds at a few positions within the EC cavity, mostly interacting with Arg^30^ and/or Asp^404^ (data not shown), two residues reported (22, 48) to coordinate the S2 site. Even when it diffused near the S2 site before complete dissociation from the transporter, there was little or no interaction with other residues (*e.g.* Tyr^108^ and Ile^111^ on TM3, Leu400 on TM10 and Ala^319^, Phe^320^, and Phe^324^ on EL4) that have been proposed (21) to line the S2 site (Fig. 4*B*).

We thoroughly examined the timing of substrate dislocation in relation to the movements of sodium ions between different sites. In Ala-bound transporter (run 5), the sodium ion at Na1 remained stably bound for the entire duration of simulations (0.5 μs) in both subunits, whereas the Na^+^ ion at Na2 moved to the EC vestibule ∼10–100 ns following the dislocation of Ala, and settled to site Na1″ (for a total duration of ∼0.2 μs in each subunit) before complete departure into the EC environment (Fig. 4*C*). Likewise, the Na^+^ ion bound to Na2 in Leu-bound transporter diffused into the EC vestibule in three (of four) subunits, whereas Leu translocated to the EC region, and in all three cases, Na2 unbinding followed that of Leu (Fig. 4*D*; see also Table 2), further highlighting the correlation between the two events. Also in harmony with the findings above, Na^+^ at Na1 either remained bound or migrated (transiently or stably) to the neighboring Na1′ site (in four of six cases) (Fig. 4*D*), where it was coordinated by Glu^290^ but never translocated to the EC vestibule/environment.

The lack of binding to S2 observed here for four of six runs (two with Ala and two with Leu) and little or no binding to most of residues that coordinate the S2 site, except to Arg^30^ and/or Asp^404^ (in two other runs with Leu) agree with studies where a stoichiometry of 1:1 was measured for Leu to LeuT and where occupancy of only S1 by various substrates co-crystallized with LeuT, under different lipid compositions, was obtained (24–26). This finding, however, does not exclude the possibility of S2 and in particular the vicinity of Arg^30^ and Asp^404^ serving as a potential substrate-binding site in other conformations of LeuT as proposed in other studies (22, 49).

##### The Gating and Structural Changes Associated with Substrate Binding

In contrast to the open conformation, in the occluded form, Ala was not released (during more than 2-μs simulations (run 7). Likewise, Leu did not escape to the EC environment in all but one (subunit B, run 8a) of the six cases (runs 8a–8c) simulated over 1.5–3 μs starting with the occluded form (Fig. 5, *E–H*). In addition, introducing the Y108F mutation did not facilitate the release of Leu within the simulated time scales of 0.5–1.4 μs (runs 9a and b).

Although the occluded form has a clearly lower tendency to release the substrate, the structural changes and gating events that led to the complete release of substrate, together with the previous events, form a complete picture of substrate- and sodium-binding mechanism: in the open form, the formation of water-mediated or direct interaction between Arg^30^ and Asp^404^ (Fig. 5*C*), as well as the rotation of Phe^253^ aromatic ring (Fig. 5*D*), lead to occluded state upon deeper insertion of the substrate (run 6a) (Fig. 5*A*) also evidenced by the decrease in the degree of EC vestibule opening (Fig. 5*B*). Conversely, in the occluded state, the reverse order of events enables substrate unbinding (Fig. 5, *E–H*), *i.e.* TM1b and 6a open first to approximately the same extent as in the open form (Fig. 5*F*), whereas the salt bridge Arg^30^–Asp^404^ starts to be destabilized (Fig. 5*G*). The disruption of the salt bridge and accompanying isomerization of the Phe^253^ side chain into its open rotameric state (Fig. 5*H*) expose the S1 site to the aqueous medium and prompt the release of Leu at ∼0.8 μs. As in the open structures, Na^+^ unbinding from Na2 via Na1″ into the EC environment, but not of Na^+^ at Na1, is possible, but only following the release of the substrate (Fig. 5*F*). Although these steps do not necessarily drive substrate release within the observed time scales, they are required as no substrate is released from the stable occluded form where TM1b and 6a maintain a degree of opening similar to or even less than that of the occluded form, with either Leu or Ala. Also, whereas TM1b and 6a were closely associated to hinder passage across the EC vestibule, Arg^30^ and Asp^404^ maintained their strong interaction, and Phe^253^ remained closed. Even when the substrate did not dislocate from S1 (runs 7, 8a-subunit A, 8b, 8c, and 9), the same type of coupling was observed to be prevalent: TM1b and 6a helices underwent occasional openings, but the Arg^30^-Asp^404^ salt bridge and the closed rotameric state of Phe^253^ were both maintained only when TM1b and 6a were closed (data not shown).

##### Hydration Promotes the Opening of the EC Vestibule

**FIGURE 6.**
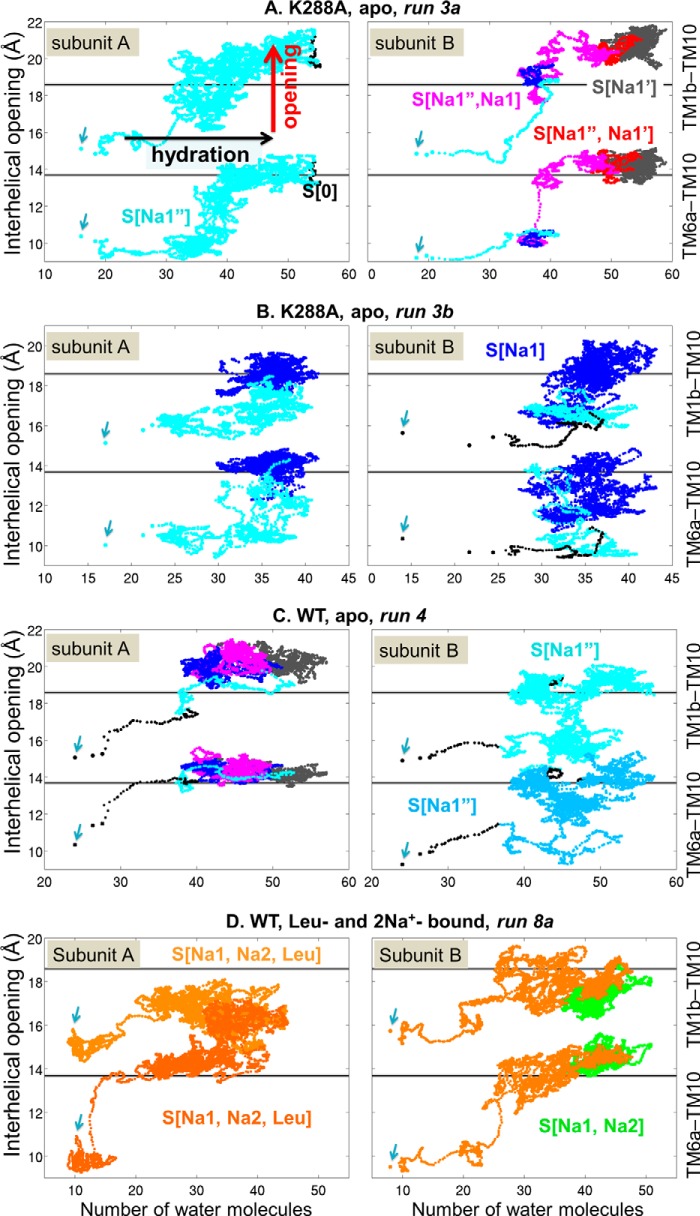
**Coupling between the hydration of the EC vestibule and the transition from occluded to open OF state.** The panels illustrate the trajectories of LeuT (WT or K288A), subunits A (*left panels*) and B (*right panels*), starting from occluded, minimally hydrated states (indicated by *arrows*). Each panel contains two sets of data points, displaying the respective interhelical distances TM1b–TM10 (based on Val^33^–Asp^401^ C^α^-C^α^ distance) and TM6a–TM10 (C^α^-C^α^ distance of Ile^245^–Ile^410^) (*right ordinate*) plotted as a function of the number of water molecules in the sodium/Leu binding pocket, defined as the region within 3 Å from ion/substrate coordinating residues as follows: (i) Na1″: Asn^21^, Ser^256^, and Ser^355^; (ii) Na1 and Na1′: Ala^22^, Asn^27^, Tyr^47^, Thr^254^, Asn^286^, and Glu^290^; (iii) Na2: Gly^20^, Val^23^, Ala^351^, Thr^354^, and Ser^355^; and (iv) S1: Ala^22^, Leu^25^, Gly^26^, Val^104^, Tyr^108^, Phe^253^, Thr^354^, Gly^258^, Ile^359^, Gly^260^, Ala^261^, and Ile^262^. The colors refer to different sodium/substrate-bound states, as labeled, *e.g.* S[0] (*black*), apo state; S[Na1″] (*cyan*), single Na^+^, bound at Na1″; S(Na1″, Na1) (*magenta*) two Na^+^ ions bound at Na1″ and Na1, etc. In *C* (*right panel*) and *D* (*left panel*), slightly different shades are used to distinguish the two trajectories.

Previous studies pointed to the role of hydration in substrate release (49), to passive water conduction of transporters in general (45), and to the distinctive hydration patterns of LeuT OF- and IF states of LeuT (48). To assess the role of hydration in the opening of the substrate-binding pocket, we thoroughly examined the number of water molecules that entered the substrate-binding pocket as a function of the level of separations of TM1b/TM6a from TM10. Trajectories initiated from the occluded conformations (runs 3–4 and 7–9) were analyzed to this end. Results (Fig. 6, *A–C*) show that prior to EC vestibule opening (ordinate), the initial event is the influx of water molecules: the number of water molecules in the EC vestibule increases from 15–20 to 30–35, which then triggers the distinctive movements of TM1b (*upper trajectory* in each panel) or TM6a (*lower trajectory*) away from TM10 by 3–4 Å. In the presence of Leu (Fig. 6*D*), the opening of the EC vestibule starts with fewer (∼15–20) water molecules. This opening further promotes the influx of water, up to ∼40 water molecules in Na1-bound LeuT and ∼55 in Na1′- and/or Na1″-bound LeuT.

## DISCUSSION

Biochemical and crystallographic data are essential to understanding the structure and mechanism of biomolecular machines such as transport systems. On the other hand, computational modeling and simulation may oftentimes be indispensible for understanding and complementing the fine details of such time-evolved processes such as the entry or exit of the substrate and co-transported ions, their timing and pathways, as explored here. Recently, a crystal structure of the dopamine transporter from *Drosophila melanogaster* has been resolved (45). This OF-open structure of dopamine transporter with a tricyclic antidepressant bound at the primary site is similar to that of the OF-open LeuT with sodium only or with tryptophan at S1. In addition, the resolved dopamine transporter structure has two sodium ions bound at the equivalent Na1 and Na2 sites, further supporting the relevance of LeuT as a model for the study of NSS. Present simulations demonstrate that starting with a known crystallized conformation of LeuT, OF-open or -occluded, the other can be spontaneously obtained, and the atomic events enabling this transition may be visualized.

Insights provided by current simulations into the mechanism of EC gating by LeuT may be summarized as follows: (i) a new sodium-binding site, Na1″, is discovered; (ii) this site serves as a first attractor for the entry of the EC Na^+^ ions to the EC vestibule; (iii) binding to Na1″ take places within tens of nanoseconds even before the opening of the EC vestibule, whereas unbinding is ∼1 order of magnitude slower; (iv) sodium at Na1″ tends to dislocate to Na1 within hundreds of nanoseconds, and the vacated Na1″ alternates between bound (to a 2nd Na^+^) and free states; (v) among the two known binding sites, Na1 exhibits a stronger affinity for sodium; (vi) sodium at Na1 often migrates to Na1′, from which it can only move back to Na1; (vii) the opening of the EC vestibule (of occluded LeuT) is prompted by an influx of water; and (viii) unbinding of Leu at S1 is succeeded by that of sodium from Na2. Finally, although the canonical function is the transport of neurotransmitters from the EC region to the IC, efflux of substrate and cations is a common phenomenon in transporters, and the observed events shed light onto possible efflux mechanisms.

**FIGURE 7.**
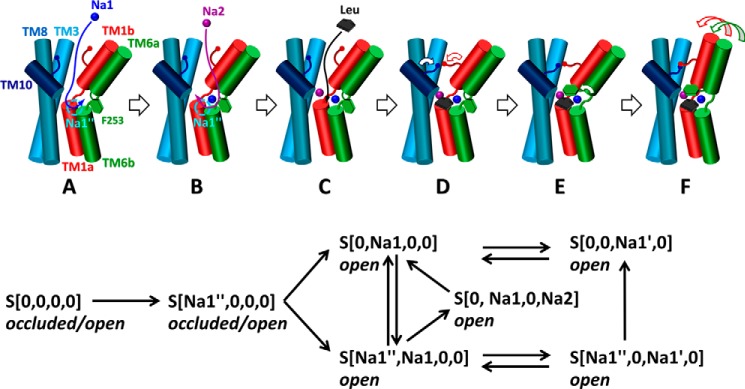
**Mechanism of sodium- and substrate-binding in the OF state of LeuT.** *A–E*, in the OF-open LeuT, a sodium ion entering the EC vestibule from the EC region first binds to the Na1 site (*A*), followed by another at Na2 (*B*) (*blue* and *purple spheres*, respectively), with the entry path for both likely to be through Na1″ site (*cyan circle*). Leucine (*black*) then enters into the EC vestibule and binds to S1 (*C*), with little or no binding to S2. Although the EC vestibule is open (*A–C*), a water-mediated or direct interaction between Arg^30^ and Asp^404^ (*blue* and *red sticks*, respectively) can take place, which, together with the isomeric rotation of the aromatic ring of Phe^253^ (*green hexagon*), enables the EC gate closure (*D*). Closure of TM1b and 6a and formation of a stable salt bridge between Arg^30^ and Asp^404^ stabilize the substrate- and sodium-loaded occluded state (*E*). The *lower panel* provides a schematic description of the initial sodium binding events along with subsequent translocations observed in the simulations.

The sequence of EC gating events drawn from this study is illustrated in Fig. 7. First, an EC sodium enters the EC vestibule and binds the Na1″ cavity. Second, it consistently translocates from Na1″ to Na1 (and may migrate from Na1 to Na1′). Third, another EC Na^+^ binds the vacated Na1″ site, before occupying the Na2 site. Na1″ thus is a first stop for sodium binding, whether the same ion translocates then to Na1 (most probable), Na1′, or Na2 (when Na1 is already occupied). Sodium binding events are consistently associated with the opening of the EC vestibule, probed by TM1b–TM10 and TM6a–TM10 distances. The exposure of the EC vestibule favors the binding of substrate (Leu/Ala) to S1. S1 is an energetically “frustrated” site located at the kinking of helices TM1 and TM6, which presents avidity for any stabilizing interaction. Substrate binding triggers a redistribution of interactions near S1, in favor of the closure of the EC vestibule, stabilized by salt bridge formation between Arg^30^ and Asp^404^ and then Phe^253^ side chain isomerization to seclude the substrate from the EC medium. Finally, TM1b and 6a close to a degree similar to the occluded OF state before further closure to take on a conformation similar to that in the IF state of LeuT.

If we further focus on sodium (un)binding and translocation events during the 20+ μs simulations, 54 transitions between different ligation states (bound to Na1, Na1′, Na1″, and/or Na2) were detected. The *lower panel* in Fig. 7 depicts these events, starting from apo LeuT. In accord with the canonical transport cycle, the rate of Na^+^ binding from the EC region was generally higher than that of unbinding. Na^+^ binding to apo LeuT was observed eight times in our simulations, all consistently to the site Na1″. The binding and unbinding rates for Na1″, based on the reciprocal of the average first passage times (40) (*t*_u→b_ = 0.018 ± 0.012 μs and *t*_b→u_ = 0.43 ± 0.177 μs) were *k*_b_ = 55/μs and *k*_u_ = 2.3/μs, Frequent exchanges between sites Na1 and Na1′ were observed, with residence times of *t*_Na1→Na1′_ = 0.173 ± 0.096 μs (average over 10 incidences) and *t*_Na1′→Na1_ = 0.060 ± 0.055 μs. The observations underscore the complexity of even sodium binding kinetics, which is often (mis)represented by a “single event,” if not by a continuum, in mathematical models.

The pathway of entry/exit of Na^+^ ions and accompanying local conformational changes are hardly detected by experiments, whereas the concerted motion of TM1b and 6a, may be probed by single molecule experiments such as FRET, or by cross-linking of cysteines introduced into both helices. Cross-linking of cysteines that can be spatially proximal because of conformational flexibility may have detectable effects on substrate uptake (*e.g.* intersubunit cross-linking of cysteines guided by simulations was observed in EAAT1 to induce significant reduction in glutamate uptake (50)). Retention of activity despite formation of cross-links would indicate that the two domains need not move independently for binding/transport to take place. If, on the contrary, there is a loss of activity, that would lend support to the role of interhelical movements in mediating transport. The role of the sites Na1, Na2, Na1″, and Na1′ in the influx/efflux properties of LeuT and transport cycle may be further assessed by single or double mutations (*e.g.* replacement by alanines) at the specific residues reported above to coordinate each of those sites. The above study thus provides testable findings on the identity and time-evolved interactions of such residues the functional role of which await further validation by biochemical or molecular biology experiments.

## Footnotes

3 The abbreviations used are:

NSS: neurotransmitter:sodium symporter
TM: transmembrane
OF: outward facing
PDB: Protein Data Bank
EC: extracellular
MD: molecular dynamics
IF: inward facing
POPE: 1-palmitoyl-2-oleoyl-phosphatidylethanolamine.

## References

[1] Richerson G. B. Wu Y. (2004) Role of the GABA transporter in epilepsy. Adv. Exp. Med. Biol. 548, 76–91 15250587 10.1007/978-1-4757-6376-8_6

[2] Andersen J. Kristensen A. S. Bang-Andersen B. Stromgaard K. (2009) Recent advances in the understanding of the interaction of antidepressant drugs with serotonin and norepinephrine transporters. Chem. Commun. (Camb.) 25, 3677–3692 19557250 10.1039/b903035m

[3] Shaffer P. L. Goehring A. Shankaranarayanan A. Gouaux E. (2009) Structure and mechanism of a Na^+^-independent amino acid transporter. Science 325, 1010–1014 19608859 10.1126/science.1176088 PMC2851542

[4] Clausen R. P. Madsen K. Larsson O. M. Frølund B. Krogsgaard-Larsen P. Schousboe A. (2006) Structure-activity relationship and pharmacology of γ-aminobutyric acid (GABA) transport inhibitors. Adv. Pharmacol. 54, 265–284 17175818 10.1016/s1054-3589(06)54011-6

[5] Hahn M. K. Blakely R. D. (2002) Monoamine transporter gene structure and polymorphisms in relation to psychiatric and other complex disorders. Pharmacogenomics J. 2, 217–235 12196911 10.1038/sj.tpj.6500106

[6] Amara S. G. Sonders M. S. (1998) Neurotransmitter transporters as molecular targets for addictive drugs. Drug Alcohol Depend. 51, 87–96 9716932 10.1016/s0376-8716(98)00068-4

[7] Fuller R. W. Wong D. T. Robertson D. W. (1991) Fluoxetine, a selective inhibitor of serotonin uptake. Med. Res. Rev. 11, 17–34 1994152 10.1002/med.2610110103

[8] Wei H. Hill E. R. Gu H. H. (2009) Functional mutations in mouse norepinephrine transporter reduce sensitivity to cocaine inhibition. Neuropharmacology 56, 399–404 18824182 10.1016/j.neuropharm.2008.09.008 PMC2666010

[9] Amara S. G. Pacholczyk T. (1991) Sodium-dependent neurotransmitter reuptake systems. Curr. Opin. Neurobiol. 1, 84–90 1688010 10.1016/0959-4388(91)90014-x

[10] Kanner B. I. Zomot E. (2008) Sodium-coupled neurotransmitter transporters. Chem. Rev. 108, 1654–1668 18393466 10.1021/cr078246a

[11] Clark J. A. Amara S. G. (1994) Stable expression of a neuronal γ-aminobutyric acid transporter, GAT-3, in mammalian cells demonstrates unique pharmacological properties and ion dependence. Mol. Pharmacol. 46, 550–557 7935337

[12] Radian R. Kanner B. I. (1983) Stoichiometry of sodium- and chloride-coupled γ-aminobutyric acid transport by synaptic plasma membrane vesicles isolated from rat brain. Biochemistry 22, 1236–1241 6838850 10.1021/bi00274a038

[13] Roux M. J. Supplisson S. (2000) Neuronal and glial glycine transporters have different stoichiometries. Neuron 25, 373–383 10719892 10.1016/s0896-6273(00)80901-0

[14] Krishnamurthy H. Gouaux E. (2012) X-ray structures of LeuT in substrate-free outward-open and apo inward-open states. Nature 481, 469–474 22230955 10.1038/nature10737 PMC3306218

[15] Singh S. K. Piscitelli C. L. Yamashita A. Gouaux E. (2008) A competitive inhibitor traps LeuT in an open-to-out conformation. Science 322, 1655–1661 19074341 10.1126/science.1166777 PMC2832577

[16] Yamashita A. Singh S. K. Kawate T. Jin Y. Gouaux E. (2005) Crystal structure of a bacterial homologue of Na^+^/Cl^−^-dependent neurotransmitter transporters. Nature 437, 215–223 16041361 10.1038/nature03978

[17] Zhou Z. Zhen J. Karpowich N. K. Goetz R. M. Law C. J. Reith M. E. Wang D. N. (2007) LeuT-desipramine structure reveals how antidepressants block neurotransmitter reuptake. Science 317, 1390–1393 17690258 10.1126/science.1147614 PMC3711652

[18] Forrest L. R. Zhang Y. W. Jacobs M. T. Gesmonde J. Xie L. Honig B. H. Rudnick G. (2008) Mechanism for alternating access in neurotransmitter transporters. Proc. Natl. Acad. Sci. U.S.A. 105, 10338–10343 18647834 10.1073/pnas.0804659105 PMC2480614

[19] Singh S. K. Yamashita A. Gouaux E. (2007) Antidepressant binding site in a bacterial homologue of neurotransmitter transporters. Nature 448, 952–956 17687333 10.1038/nature06038

[20] Quick M. Winther A. M. Shi L. Nissen P. Weinstein H. Javitch J. A. (2009) Binding of an octylglucoside detergent molecule in the second substrate (S2) site of LeuT establishes an inhibitor-bound conformation. Proc. Natl. Acad. Sci. U.S.A. 106, 5563–5568 19307590 10.1073/pnas.0811322106 PMC2667088

[21] Quick M. Shi L. Zehnpfennig B. Weinstein H. Javitch J. A. (2012) Experimental conditions can obscure the second high-affinity site in LeuT. Nat. Struct. Mol. Biol. 19, 207–211 22245968 10.1038/nsmb.2197 PMC3272158

[22] Shi L. Quick M. Zhao Y. Weinstein H. Javitch J. A. (2008) The mechanism of a neurotransmitter:sodium symporter: inward release of Na^+^ and substrate is triggered by substrate in a second binding site. Mol. Cell 30, 667–677 18570870 10.1016/j.molcel.2008.05.008 PMC2826427

[23] Zhao Y. Terry D. S. Shi L. Quick M. Weinstein H. Blanchard S. C. Javitch J. A. (2011) Substrate-modulated gating dynamics in a Na^+^-coupled neurotransmitter transporter homologue. Nature 474, 109–113 21516104 10.1038/nature09971 PMC3178346

[24] Piscitelli C. L. Krishnamurthy H. Gouaux E. (2010) Neurotransmitter/sodium symporter orthologue LeuT has a single high-affinity substrate site. Nature 468, 1129–1132 21179170 10.1038/nature09581 PMC3079577

[25] Wang H. Gouaux E. (2012) Substrate binds in the S1 site of the F253A mutant of LeuT, a neurotransmitter sodium symporter homologue. EMBO Rep. 13, 861–866 22836580 10.1038/embor.2012.110 PMC3432802

[26] Wang H. Elferich J. Gouaux E. (2012) Structures of LeuT in bicelles define conformation and substrate binding in a membrane-like context. Nat. Struct. Mol. Biol. 19, 212–219 22245965 10.1038/nsmb.2215 PMC3322350

[27] Lester H. A. Cao Y. Mager S. (1996) Listening to neurotransmitter transporters. Neuron 17, 807–810 8938113 10.1016/s0896-6273(00)80213-5

[28] Scholze P. Freissmuth M. Sitte H. H. (2002) Mutations within an intramembrane leucine heptad repeat disrupt oligomer formation of the rat GABA transporter 1. J. Biol. Chem. 277, 43682–43690 12223478 10.1074/jbc.M205602200

[29] Bartholomäus I. Milan-Lobo L. Nicke A. Dutertre S. Hastrup H. Jha A. Gether U. Sitte H. H. Betz H. Eulenburg V. (2008) Glycine transporter dimers: evidence for occurrence in the plasma membrane. J. Biol. Chem. 283, 10978–10991 18252709 10.1074/jbc.M800622200 PMC4503265

[30] Sorkina T. Doolen S. Galperin E. Zahniser N. R. Sorkin A. (2003) Oligomerization of dopamine transporters visualized in living cells by fluorescence resonance energy transfer microscopy. J. Biol. Chem. 278, 28274–28283 12746456 10.1074/jbc.M210652200

[31] Norgaard-Nielsen K. Norregaard L. Hastrup H. Javitch J. A. Gether U. (2002) Zn^2+^ site engineering at the oligomeric interface of the dopamine transporter. FEBS Lett. 524, 87–91 12135746 10.1016/s0014-5793(02)03008-9

[32] Faham S. Watanabe A. Besserer G. M. Cascio D. Specht A. Hirayama B. A. Wright E. M. Abramson J. (2008) The crystal structure of a sodium galactose transporter reveals mechanistic insights into Na^+^/sugar symport. Science 321, 810–814 18599740 10.1126/science.1160406 PMC3654663

[33] Hénin J. Shinoda W. Klein M. L. (2008) United-atom acyl chains for CHARMM phospholipids. J. Phys. Chem. B. 112, 7008–7015 18481889 10.1021/jp800687p PMC8451178

[34] Best R. B. Zhu X. Shim J. Lopes P. E. Mittal J. Feig M. Mackerell A. D. Jr. (2012) Optimization of the additive CHARMM all-atom protein force field targeting improved sampling of the backbone phi, psi and side-chain χ_1_ and χ_2_ dihedral angles. J. Chem. Theory Comput. 8, 3257–3273 23341755 10.1021/ct300400x PMC3549273

[35] Buck M. Bouguet-Bonnet S. Pastor R. W. MacKerell A. D. Jr. (2006) Importance of the CMAP correction to the CHARMM22 protein force field: dynamics of hen lysozyme. Biophys. J. 90, L36–L38 16361340 10.1529/biophysj.105.078154 PMC1367299

[36] Phillips J. C. Braun R. Wang W. Gumbart J. Tajkhorshid E. Villa E. Chipot C. Skeel R. D. Kalé L. Schulten K. (2005) Scalable molecular dynamics with NAMD. J. Comput. Chem. 26, 1781–1802 16222654 10.1002/jcc.20289 PMC2486339

[37] Shaw D. E. Deneroff M. M. Dror R. O. Kuskin J. S. Larson R. H. Salmon J. K. Young C. Batson B. Bowers K. J. Chao J. C. Eastwood M. O. Gagliardo J. Grossman J. P. Ho C. R. Ierardi D. J. Kolossváry I. Klepeis J. L. Layman T. McLeavey C. Moraes M. A. Mueller R. Priest E. C. Shan Y. Spengler J. Theobald M. Towles B. Wang S. C. (2007) Anton, a special-purpose machine for molecular dynamics simulation. ACM SIGARCH Computer Architecture News 35, 1–12

[38] Humphrey W. Dalke A. Schulten K. (1996) VMD: visual molecular dynamics. J. Mol. Graph. 14, 33–38 8744570 10.1016/0263-7855(96)00018-5

[39] Zhao C. Stolzenberg S. Gracia L. Weinstein H. Noskov S. Shi L. (2012) Ion-controlled conformational dynamics in the outward-open transition from an occluded state of LeuT. Biophys. J. 103, 878–888 23009837 10.1016/j.bpj.2012.07.044 PMC3433624

[40] Gur M. Zomot E. Bahar I. (2013) Global motions exhibited by proteins in micro- to milliseconds simulations concur with anisotropic network model predictions. J. Chem. Phys. 139, 121912 24089724 10.1063/1.4816375 PMC3739829

[41] Quick M. Yano H. Goldberg N. R. Duan L. Beuming T. Shi L. Weinstein H. Javitch J. A. (2006) State-dependent conformations of the translocation pathway in the tyrosine transporter Tyt1, a novel neurotransmitter:sodium symporter from *Fusobacterium nucleatum*. J. Biol. Chem. 281, 26444–26454 16798738 10.1074/jbc.M602438200

[42] Androutsellis-Theotokis A. Goldberg N. R. Ueda K. Beppu T. Beckman M. L. Das S. Javitch J. A. Rudnick G. (2003) Characterization of a functional bacterial homologue of sodium-dependent neurotransmitter transporters. J. Biol. Chem. 278, 12703–12709 12569103 10.1074/jbc.M206563200

[43] Kantcheva A. K. Quick M. Shi L. Winther A. M. Stolzenberg S. Weinstein H. Javitch J. A. Nissen P. (2013) Chloride binding site of neurotransmitter sodium symporters. Proc. Natl. Acad. Sci. U.S.A. 110, 8489–8494 23641004 10.1073/pnas.1221279110 PMC3666746

[44] Tavoulari S. Rizwan A. N. Forrest L. R. Rudnick G. (2011) Reconstructing a chloride-binding site in a bacterial neurotransmitter transporter homologue. J. Biol. Chem. 286, 2834–2842 21115480 10.1074/jbc.M110.186064 PMC3024779

[45] Penmatsa A. Wang K. H. Gouaux E. (2013) X-ray structure of dopamine transporter elucidates antidepressant mechanism. Nature 503, 85–90 24037379 10.1038/nature12533 PMC3904663

[46] Forrest L. R. Tavoulari S. Zhang Y. W. Rudnick G. Honig B. (2007) Identification of a chloride ion binding site in Na^+^/Cl-dependent transporters. Proc. Natl. Acad. Sci. U.S.A. 104, 12761–12766 17652169 10.1073/pnas.0705600104 PMC1937540

[47] Zomot E. Bendahan A. Quick M. Zhao Y. Javitch J. A. Kanner B. I. (2007) Mechanism of chloride interaction with neurotransmitter:sodium symporters. Nature 449, 726–730 17704762 10.1038/nature06133

[48] Zhao Y. Quick M. Shi L. Mehler E. L. Weinstein H. Javitch J. A. (2010) Substrate-dependent proton antiport in neurotransmitter:sodium symporters. Nat. Chem. Biol. 6, 109–116 20081826 10.1038/nchembio.284 PMC2808765

[49] Cheng M. H. Bahar I. (2013) Coupled global and local changes direct substrate translocation by neurotransmitter-sodium symporter ortholog LeuT. Biophys. J. 105, 630–639 23931311 10.1016/j.bpj.2013.06.032 PMC3736663

[50] Jiang J. Shrivastava I. H. Watts S. D. Bahar I. Amara S. G. (2011) Large collective motions regulate the functional properties of glutamate transporter trimers. Proc. Natl. Acad. Sci. U.S.A. 108, 15141–15146 21876140 10.1073/pnas.1112216108 PMC3174670

